# When mild impairment is not safe: mini-mental state examination scores of ≤21 are associated with emergency response failure

**DOI:** 10.3389/fnagi.2026.1916712

**Published:** 2026-08-07

**Authors:** Howard Kim, Caitlyn Parker, Jennifer Jurado Severance, April Wiechmann

**Affiliations:** 1 UNT Health Fort Worth Texas College of Osteopathic Medicine, Fort Worth, TX, United States; 2 Department of Internal Medicine, University of North Texas Health Fort Worth, Fort Worth, TX, United States

**Keywords:** dementia, emergency response, executive function, functional assessment, independent living, mild cognitive impairment, mini-mental state examination, safety screening

## Abstract

**Introduction:**

Unrecognized emergency-response deficits contribute to preventable harm and loss of independence in older adults with cognitive decline. Although the Mini-Mental State Examination (MMSE) is widely used to assess global cognition, clinicians often use MMSE scores to inform judgments about everyday functioning. However, the relationship between MMSE performance and emergency-response ability remains unclear. The Test of Executive Functioning in an Emergency (TEFE) is a brief, performance-based assessment that evaluates a patient’s ability to relay critical information and contact emergency services. This study examined whether an MMSE threshold was associated with failure on TEFE 911 tasks.

**Methods:**

A retrospective analysis included 905 patients from a university-affiliated memory clinic with Alzheimer’s disease, vascular dementia, mixed dementia, mild cognitive impairment, or normal cognition. Associations between MMSE scores and TEFE 911 knowledge and behavioral task failure were examined using correlation, adjacent-score comparisons, receiver operating characteristic (ROC) analyses, Youden’s *J*, and multivariable logistic regression adjusted for demographic and diagnostic covariates.

**Results:**

MMSE and TEFE scores were moderately correlated (*r* = 0.596, *p* < 0.0001). Failure rates were higher at MMSE scores of 21 than 22. ROC analyses demonstrated good discrimination for 911 task failure (AUC = 0.860–0.867). An MMSE cutoff of ≤21 yielded 61.9% sensitivity and 88.9% specificity for failure on 911 tasks and remained independently associated with failure after adjustment (adjusted OR = 3.71, 95% CI: 1.82–7.56).

**Discussion:**

Patients with MMSE scores ≤21 may warrant further performance-based assessment of emergency-response ability. Prospective studies should determine whether this threshold predicts real-world safety outcomes.

## 1 Introduction

Patients who exhibit early symptoms of cognitive decline are often referred for neuropsychological evaluation in outpatient clinic settings where standardized assessments are used to evaluate a patient’s capacity to perform activities of daily living (ADLs) and instrumental activities of daily living (IADLs). Clinical assessment helps to determine independence with medication management, financial decision-making, and ability to travel independently (Wiechmann et al., 2015). While dementia progression ultimately leads to widespread impairment in basic ADLs (Mioshi et al., 2007; Mioshi and Hodges, 2009), early stages of disease, particularly in conditions such as Alzheimer’s disease (AD) and frontotemporal dementia (FTD), disproportionately affect IADLs (Chatzidimitriou et al., 2023), which place greater demands on higher-order cognitive processes, particularly executive functioning. Accordingly, early deficits in neurodegenerative disease often manifest as executive dysfunction, characterized by impairments in planning, sequencing, and goal-directed behavior (Miyake and Friedman, 2012). In AD, executive dysfunction has been associated with clinically significant outcomes, including medication nonadherence, wandering, and inadequate responses to emergencies (Royall et al., 2007). These impairments likely reflect early neuropathological changes in prefrontal and medial temporal networks that support the integration, monitoring, and execution of complex real-world behaviors (Chatzidimitriou et al., 2023).

These safety vulnerabilities often become the tipping point for transitions from independent living to supervised care, a shift that carries profound consequences for patients who view remaining in their own homes as central to their well-being and identity (Forsund et al., 2018). This tension between safety and independence need not result in an all-or-nothing decision. Environmental modifications and assistive technologies, such as home monitoring systems, medical alert devices, and simplified communication tools that enable direct contact with family or emergency services, can serve as intermediate supports that preserve independence while mitigating risk (Gettel et al., 2021). Accurately identifying the nature and degree of a patient’s safety deficits is therefore essential, not only to determine the appropriate level of care, but also to guide individualized safety planning that aligns with both the patient’s and family’s goals.

Current clinical tools, however, may not adequately identify patients whose cognitive impairment has begun to compromise emergency-response abilities. While the Mini-Mental State Examination (MMSE; Folstein et al., 2001) and Trail Making Test-B provide valuable insights into global cognition and attention (MacPherson et al., 2019), they are not designed to directly assess emergency-response abilities required for safe independent living. A patient might perform adequately on standard screeners yet lack the ability to call 911, recite their address, or operate a phone, all of which are skills essential for independent living. Other assessments such as the Direct Assessment of Functional Status-Revised (DAFS-R), the Performance Assessment of Self-Care Skills (PASS), and the Pillbox Test address IADLs (McDougall et al., 2010) but do not directly evaluate emergency preparedness. The Clinical Dementia Rating (CDR) scale can inform decisions such as driving safety (Iverson et al., 2010), yet no widely adopted tool directly assesses emergency response abilities relevant to independent living.

The ability to recognize and respond to emergencies is a critical component of independent functioning, and unsafe activities and living conditions are common concerns in older adults with dementia (Amjad et al., 2016). Given that 58.7% of older adults with probable dementia have been reported to be either undiagnosed or unaware of their diagnosis (Amjad et al., 2018), and that primary care visits are often constrained by limited time and competing clinical demands (Bradford et al., 2009), there is a pressing need for a brief functional measure that enhances existing screening practices. The Test of Executive Functioning in an Emergency (TEFE; Wiechmann et al., 2015) was developed by clinical staff at UNT Health Fort Worth to address this gap. TEFE is a brief, performance-based tool that evaluates a patient’s ability to relay critical information and contact emergency services.

Although prior work has examined the relationship between cognitive impairment and functional decline, less is known about how commonly used global cognitive screening scores relate to specific emergency-response abilities. This distinction is clinically important because emergency response requires more than general cognitive intactness; patients must recognize a crisis, retrieve the appropriate emergency number, and carry out the behavior of initiating a call. The present study differs from prior work by examining whether MMSE scores are associated with failure on specific TEFE 911 tasks, which directly assess knowledge of and ability to contact emergency services. Because the MMSE is already widely used in clinical settings, determining whether patients with lower MMSE scores are more likely to fail TEFE emergency-response tasks may help clinicians identify individuals who warrant additional performance-based functional safety assessment. Accordingly, the primary aim of this study was to examine the relationship between MMSE scores and TEFE performance, and determine whether lower MMSE scores were associated with failure on TEFE emergency-response tasks.

## 2 Methods

### 2.1 Sample

This retrospective cross-sectional study reviewed clinical records from 905 patients evaluated at the Center for Older Adults at UNT Health Fort Worth. The study protocol was reviewed and approved by the North Texas Regional Institutional Review Board. Participants were classified into one of six diagnostic groups: Alzheimer’s disease (AD), vascular dementia (VD), mixed dementia, mild cognitive impairment (MCI)—nonamnestic type, MCI—amnestic type, and normal cognition. All patients were 60 years of age or older.

The sample included mild dementia cases only—Alzheimer’s disease (AD; *n* = 101), vascular dementia (VD; *n* = 69), and mixed dementia (*n* = 75)—as well as nonamnestic MCI (*n* = 474), amnestic MCI (*n* = 116), and normal cognition (*n* = 73). Dementia severity (mild, moderate, severe) was rated by the evaluating clinician based on the comprehensive diagnostic workup, including clinical history and neuropsychological testing, and, when available, neuroimaging and laboratory findings. Only individuals classified as mild were included in the dementia groups for the primary analysis. This decision reflects the clinical population most likely to undergo early evaluation in memory clinics and be considered for continued independent living. Demographic characteristics by group are summarized in Table 1.

**Table 1:** Demographic characteristics by group.

Diagnosis	Count	Mean age	SD age	Mean education	SD education	% Female	% White
Cognitively normal	73	74.78	6.93	15.03	2.71	58.9	83.56
MCI-amnestic	116	75.85	7.03	13.92	2.55	61.21	79.31
MCI- non-amnestic	474	74.38	7.68	14.86	2.63	60.97	83.97
Mild AD	101	79.37	6.74	15.02	6.75	61.39	82.18
Mild mixed	75	76.72	7.65	13.64	3.48	57.33	82.67
Mild VD	69	78.93	14.14	14.14	2.95	53.62	62.32

### 2.2 Instruments

The patients completed a comprehensive neuropsychological battery consisting of 25 cognitive measures assessing executive functioning, memory, language, problem solving, attention, learning, motor functioning, and psychological functioning. The battery included the TEFE and the MMSE.

The TEFE is a brief, performance-based measure designed to assess emergency readiness through both knowledge- and behavior-based items rooted in procedural and declarative memory. To standardize the behavioral assessment, a large-button desk phone was provided for tasks involving dialing numbers. This model was chosen for its accessibility, especially in consideration of older adults with arthritis or dexterity limitations. Although the phone differed from those used in many homes, it minimized motor barriers while allowing standardized assessment of emergency dialing. Patients were asked whether they had difficulty using their own phone, and caregivers were queried for collateral reports of phone use at home (Wiechmann et al., 2015). When possible, patients were allowed and encouraged to use their personal phone to complete the task.

The MMSE is a widely used screening exam that assesses orientation, attention, language, memory, and visuospatial reasoning. It involves a series of questions and tasks but does not require any specialized equipment beyond a piece of paper and a writing utensil.

### 2.3 Procedure

The TEFE consists of six items designed to assess practical emergency functioning through both knowledge and behavioral components. The knowledge-based items require the individual to state the correct emergency number (911), recall their home address, and provide an emergency contact phone number. These are paired with corresponding behavioral tasks, including physically dialing 911 using a phone, writing their home address, and writing the emergency contact’s phone number. Each item was administered independently and scored in real time, requiring both cognitive recall and functional output in order to approximate real-world emergency demands. Each correct response was awarded one point, yielding a total score ranging from 0 to 6, with higher scores reflecting better functional emergency capacity. Responses were scored as correct only if completed independently and without prompting. A standardized version of the TEFE is available for clinical use via the QR code provided below.

The MMSE consists of 11 components scored out of 30 total points. The scores were analyzed both as a continuous variable and categorically using standard ranges: 24–30 (no impairment), 18–23 (mild impairment), 10–17 (moderate impairment), and 0–9 (severe impairment).

### 2.4 Primary outcome: 911 emergency response failure

The two 911 items were selected as the primary outcomes for this study because they most directly represent the core emergency-response behavior of identifying and contacting emergency services. Unlike home-address and emergency contact items, which may be affected by recent relocation, reliance on stored contacts, or individual communication practices, knowledge of 911 and the ability to initiate a 911 call represent relatively standardized expectations in the United States.

Item-level performance also showed no failures on either 911 item among cognitively normal participants, whereas the remaining TEFE items demonstrated greater variability. This pattern suggests that the 911 tasks represent highly overlearned and consistently preserved functional expectations. Because these items probe emergency preparedness and reflect preserved procedural and semantic knowledge, failure on either task may indicate functional safety risk. For this reason, failure on either the 911 knowledge item or 911 behavioral item was used as the primary outcome. Because this analysis was retrospective, the selection of the 911 outcomes should be considered exploratory; future prospective studies should pre-specify the primary TEFE outcome.

### 2.5 Statistical analyses

Of 908 records initially reviewed, three were missing both TEFE 911 knowledge and behavioral scores and were excluded from 911-task analyses, leaving 905 observations. Patients missing MMSE or covariate values were excluded only from analyses requiring those variables. Multivariable models were performed using complete cases, and no missing values were imputed.

Pearson correlation was used to examine the association between MMSE total score and TEFE total score. Scatterplots were visually inspected to confirm an approximately linear relationship between MMSE and TEFE scores. Logistic regression was then used to evaluate whether MMSE scores predicted failure on the TEFE 911 knowledge and behavioral tasks, treating failure on each task as an indicator of functional safety risk.

Because clinical decisions may be influenced by small differences in MMSE score, failure rates between adjacent MMSE values, including 21 versus 22, were compared using Fisher’s Exact Tests.

Predictive value analyses and receiver operating characteristic (ROC) analyses were used to assess sensitivity and specificity across MMSE scores and to identify an MMSE cutoff associated with TEFE 911-task failure. Youden’s *J* statistic was used to identify the cutoff that optimized the balance between sensitivity and specificity. A positive screen was defined as an MMSE score at or below each candidate cutoff.

To address potential confounding, multivariable logistic regression was performed with failure on either TEFE 911 task as the dependent variable. MMSE was first examined as a continuous predictor. A second model examined MMSE ≤21 versus >21, as identified by the ROC/Youden analysis. Models were adjusted for available demographic and clinical covariates, including age, education, sex, race, and diagnostic group. Adjusted odds ratios with 95% confidence intervals were calculated. Standardized measures of sensory impairment, motor limitations, telephone familiarity, and language barriers were not available in the retrospective dataset and therefore could not be included as covariates.

All analyses were conducted using R version 4.5.1.

## 3 Results

### 3.1 Diagnostic group differences

Patients with Alzheimer’s disease demonstrated the highest failure rates on both the knowledge and behavioral 911 tasks, followed by patients with mixed dementia and vascular dementia (Table 2). Patients with MCI showed failure rates below 6% on both 911 tasks, whereas cognitively normal individuals showed no failures on either task.

**Table 2:** Failure rates per TEFE question across the different groups.

Diagnosis	911 knowledge failure (%)	911 behavioral failure (%)	Fail knowledge address (%)	Fail behavioral address (%)	Fail knowledge of other phone # (%)	Fail behavioral of other phone # (%)
Cognitively normal	0	0	1.4	4.1	20.5	2.7
MCI-amnestic	5.2	5.2	9.5	7.8	50	12.1
MCI- non-amnestic	1.3	1.5	5.3	4.4	35.9	5.7
Mild AD	24.1	27	34.3	38	78.8	43.1
Mild mixed	12.8	10.3	23.1	25.6	61.5	28.2
Mild VD	8.5	7	28.2	29.6	60.6	29.6

### 3.2 Association between MMSE and TEFE performance

MMSE total score was moderately correlated with TEFE total score (*r* = 0.596, *p* < 0.0001). Lower global cognitive performance was associated with poorer emergency-related functional performance.

### 3.3 911 task failure across MMSE scores

**Figure 1:**
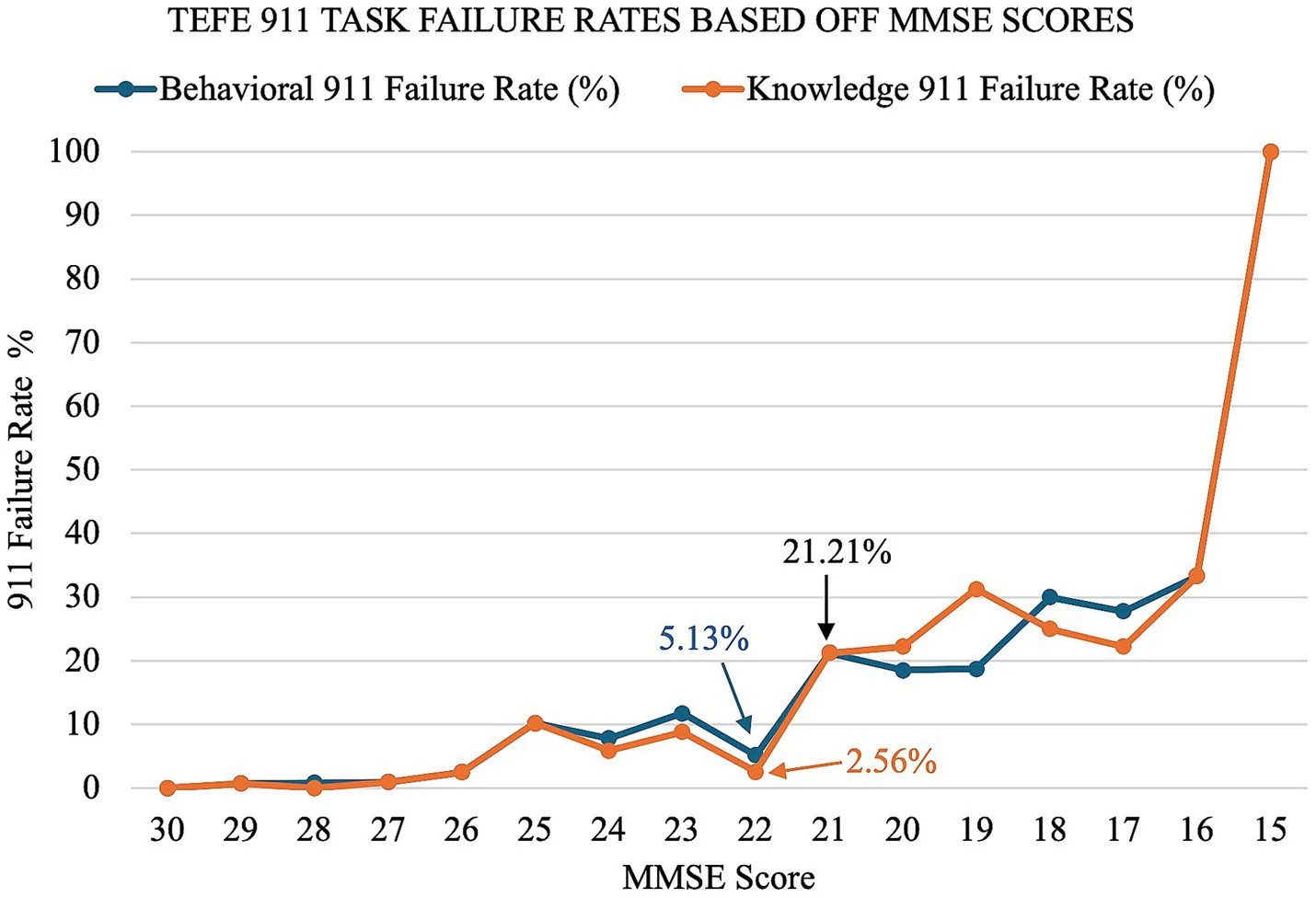
TEFE 911 knowledge and behavioral task failure rates by MMSE score.

A marked increase in TEFE 911 task failure was observed between MMSE scores of 22 and 21, as illustrated in Figure 1. Behavioral-task failure occurred in 21.21% of patients scoring 21 compared with 5.13% of those scoring 22, corresponding to a risk ratio of 4.13 (Fisher’s exact test, *p* = 0.0378). Similarly, knowledge-task failure occurred in 21.21% of patients scoring 21 compared with 2.56% of those scoring 22, corresponding to a risk ratio of 8.29 (Fisher’s exact test, *p* = 0.0202).

### 3.4 ROC analysis and discrimination

ROC analyses were performed to evaluate the ability of the MMSE to discriminate patients with TEFE 911-task failure. Emergency response failure was examined using three outcomes: failure on the TEFE 911 knowledge task, failure on the TEFE 911 behavioral task, and failure on either task.

The MMSE demonstrated strong discrimination for identifying failure on the 911 knowledge task, with an area under the curve (AUC) of 0.867 (95% CI, 0.826–0.908). Similarly, the MMSE demonstrated discrimination for failure on the 911 behavioral task, with an AUC of 0.860 (95% CI, 0.818–0.902). When emergency response failure was defined as failure on either the knowledge or behavioral task, the MMSE continued to demonstrate good performance, with an AUC of 0.862 (95% CI, 0.822–0.902).

### 3.5 Cutoff optimization using Youden’s *J* index

Sensitivity and specificity were calculated across MMSE cutoff scores using failure on either TEFE 911 task as the primary outcome. Youden’s *J* statistic was used to identify the cutoff that optimized the balance between sensitivity and specificity. The highest Youden’s *J* value was observed at an MMSE cutoff of ≤21.

At an MMSE cutoff of ≤21, sensitivity for identifying emergency response failure was 61.9% and specificity was 88.9%, corresponding to a Youden’s *J* value of 0.508. Across the examined cutoffs, lower MMSE thresholds were associated with progressively higher specificity and reduced sensitivity, reflecting the expected tradeoff between false-positive and false-negative classification (Table 3).

**Table 3:** Sensitivity, specificity, and Youden’s *J* by MMSE cutoff score.

MMSE cutoff	Sensitivity (%)	Specificity (%)	Youden’s *J*
≤22	65.1	84.5	0.496
≤21	61.9	88.9	0.508
≤20	50.8	92.1	0.429
≤19	41.3	94.6	0.359

### 3.6 Multivariable logistic regression

Multivariable logistic regression was performed to determine whether the association between MMSE ≤21 and TEFE 911-task failure persisted after adjustment for available demographic and clinical covariates. After adjustment for age, education, sex, race, and diagnostic group, MMSE ≤21 remained significantly associated with failure on either 911 task. Patients with MMSE scores ≤21 had higher odds of failing at least one 911 task compared with those scoring above 21 (adjusted OR = 3.71, 95% CI: 1.82–7.56, *p* = 0.0003). Older age and diagnostic group were also significantly associated with 911-task failure, whereas education, sex, and race were not.

## 4 Discussion

This study examined the relationship between MMSE scores and emergency-response task performance on the TEFE. Patients with MMSE scores of ≤21 were significantly more likely to fail performance-based emergency-response tasks than those with higher MMSE scores. This pattern was supported by adjacent-score comparisons between MMSE scores of 21 and 22; however, these findings should be interpreted as group-level differences in observed task performance rather than evidence that a one-point MMSE change within an individual necessarily reflects a true change in emergency-response capacity.

This threshold has important implications for how clinicians interpret “mild impairment.” Although MMSE scores of 18–23 are often grouped together, our findings suggest that this range is not clinically equivalent in terms of functional safety. Patients near this cutoff may present as socially intact and conversationally fluent, which can obscure underlying deficits in real-world functioning (Amjad et al., 2016). The ability to engage appropriately in a clinical encounter does not reliably predict the ability to perform under the cognitive demands of a real emergency, where a patient must recognize a crisis, retrieve the correct response, and communicate effectively under pressure. In a prior prospective study of 139 cognitively impaired older adults living alone, approximately 22% experienced harm requiring emergency services, with such events defined as injuries or property loss resulting from self-neglect or disorientation (Tierney et al., 2004). Notably, individuals who experienced harm in this cohort had mean MMSE scores of 21.4, closely aligning with the threshold identified in the present study. These findings are clinically relevant because executive dysfunction has been linked to IADL impairment in both mild cognitive impairment and Alzheimer’s disease, suggesting that real-world functional limitations may emerge even when impairment appears mild (Marshall et al., 2011). Without a targeted functional measure, this gap is easily missed.

The present findings identify MMSE ≤21 as a clinically meaningful threshold at which additional functional safety assessment may be warranted. At this threshold, the MMSE demonstrated moderate sensitivity and high specificity for identifying failure on either TEFE 911 task, suggesting that patients scoring at or below 21 are more likely to have emergency-response deficits on performance-based testing. This association also persisted after adjustment for available demographic and clinical covariates, including age, education, sex, race, and diagnostic group. This finding is clinically relevant because the MMSE is heavily weighted toward orientation and language items and has been criticized for limited sensitivity to executive functioning deficits, which are central to emergency response ability (Nyenhuis and Reckow, 2023). Prior work examining MMSE cutoff performance has also emphasized that optimal thresholds depend on the desired balance between sensitivity and specificity and that the MMSE has limitations in detecting early cognitive impairment (Salis et al., 2023). In this context, MMSE ≤21 should not be interpreted as a standalone measure of emergency-response ability, but rather as a potential point at which clinicians may consider additional performance-based safety assessment.

At the same time, the MMSE’s association with TEFE failure suggests that global cognitive decline still captures downstream functional risk once cognitive impairment reaches a level associated with broader functional decline. Although the MMSE is less sensitive to subtle executive deficits and may miss functional impairment at higher score ranges, lower MMSE scores may reflect broader cognitive compromise that increases the likelihood of real-world safety deficits. By the time a patient scores at or below 21, these processes may be substantially compromised, even if the MMSE never explicitly tests them. In this sense, lower MMSE scores may serve as a practical indicator of functional safety risk, allowing clinicians to identify patients who may benefit from more direct performance-based assessment without requiring additional cognitive screening.

These findings have direct clinical implications, but they should be interpreted cautiously. An MMSE score of ≤21 can serve as a practical point at which clinicians consider whether additional functional safety assessment is warranted, particularly in settings where visit time is limited and comprehensive neuropsychological evaluation is not immediately feasible. Primary care providers face well-documented barriers to early detection of cognitive impairment, including the time, training, and follow-up required for commonly used cognitive assessments, which can be difficult to implement in busy clinical workflows (Bradford et al., 2009). Because primary care practitioners often serve as the first clinicians to recognize cognitive impairment and make most initial dementia diagnoses (Siddiqui et al., 2023), missed or delayed detection in this setting has important consequences for early safety planning. This concern is reinforced by population-based evidence showing that many older adults with dementia report no history of clinical cognitive evaluation by a physician (Kotagal et al., 2015). In this context, a simple and actionable threshold becomes especially valuable.

Rather than serving as a standalone decision rule, MMSE ≤21 may prompt consideration of a brief performance-based emergency-response assessment such as the TEFE. This proposed approach uses an MMSE score of ≤21 to identify patients who may benefit from more direct evaluation of emergency-response ability while recognizing that TEFE performance should be interpreted alongside clinical history, caregiver reports, sensory and motor functioning, living environment, and other functional measures when considering supervision needs, assistive technology, or living arrangements. Because this workflow has not been prospectively evaluated, it should not yet be considered a validated clinical algorithm.

Several limitations should be considered when interpreting these findings. The retrospective design and single-center sample limit causal inference and generalizability. The sample was drawn from a university-affiliated memory clinic and included individuals with normal cognition, mild cognitive impairment, and mild dementia. This focus was intentional because these populations represent patients in whom questions regarding emergency-response ability and functional independence most commonly arise. Accordingly, the identified MMSE threshold should not be extrapolated to more advanced stages of dementia, where supervision needs and functional impairment are often already clinically apparent and different clinical questions predominate. The findings may also not generalize to unselected primary care populations, individuals evaluated in other healthcare systems, or populations that differ in demographic, cultural, linguistic, or educational characteristics. In addition, emergency-response procedures and telephone technologies vary across regions and countries, which may influence TEFE performance and should be considered when interpreting these findings.

The TEFE itself also requires broader psychometric and external validation. It was developed and studied at the same institution from which the present sample was drawn, and the current findings therefore do not establish reproducibility across independent centers. Independent replication, inter-rater reliability, and test–retest reliability have not yet been established across diverse clinical, linguistic, and cultural populations. Performance may also vary according to familiarity with telephone technology, local emergency-response systems, sensory limitations, motor limitations, and living environment. Accordingly, the present findings should not be interpreted as supporting widespread implementation of the TEFE without additional validation.

Future studies should examine whether TEFE failure at a given MMSE score predicts downstream adverse events such as delayed emergency response, injury at home, institutionalization, or loss of independent living. Prospective studies should also evaluate whether targeted interventions following a failed TEFE, such as caregiver coaching, simplified phone setup, medical-alert technology, or environmental modifications, reduce real-world safety incidents over time. Future validation work should pre-specify the TEFE outcome of interest and examine performance across diverse clinical settings, languages, emergency-response systems, and telephone technologies.

## 5 Conclusion

In summary, MMSE scores of ≤21 were associated with an increased likelihood of failure on TEFE 911 emergency-response tasks. These findings suggest that MMSE ≤21 may serve as a practical point at which clinicians consider additional performance-based assessment of emergency-response ability. Because this study was cross-sectional and conducted within a single memory-clinic sample, the proposed threshold should be considered hypothesis-generating. Prospective studies are needed to determine whether MMSE ≤21 and TEFE performance predict real-world safety outcomes, including emergency incidents, injury, institutionalization, or loss of independent living.

**Figure 2:**
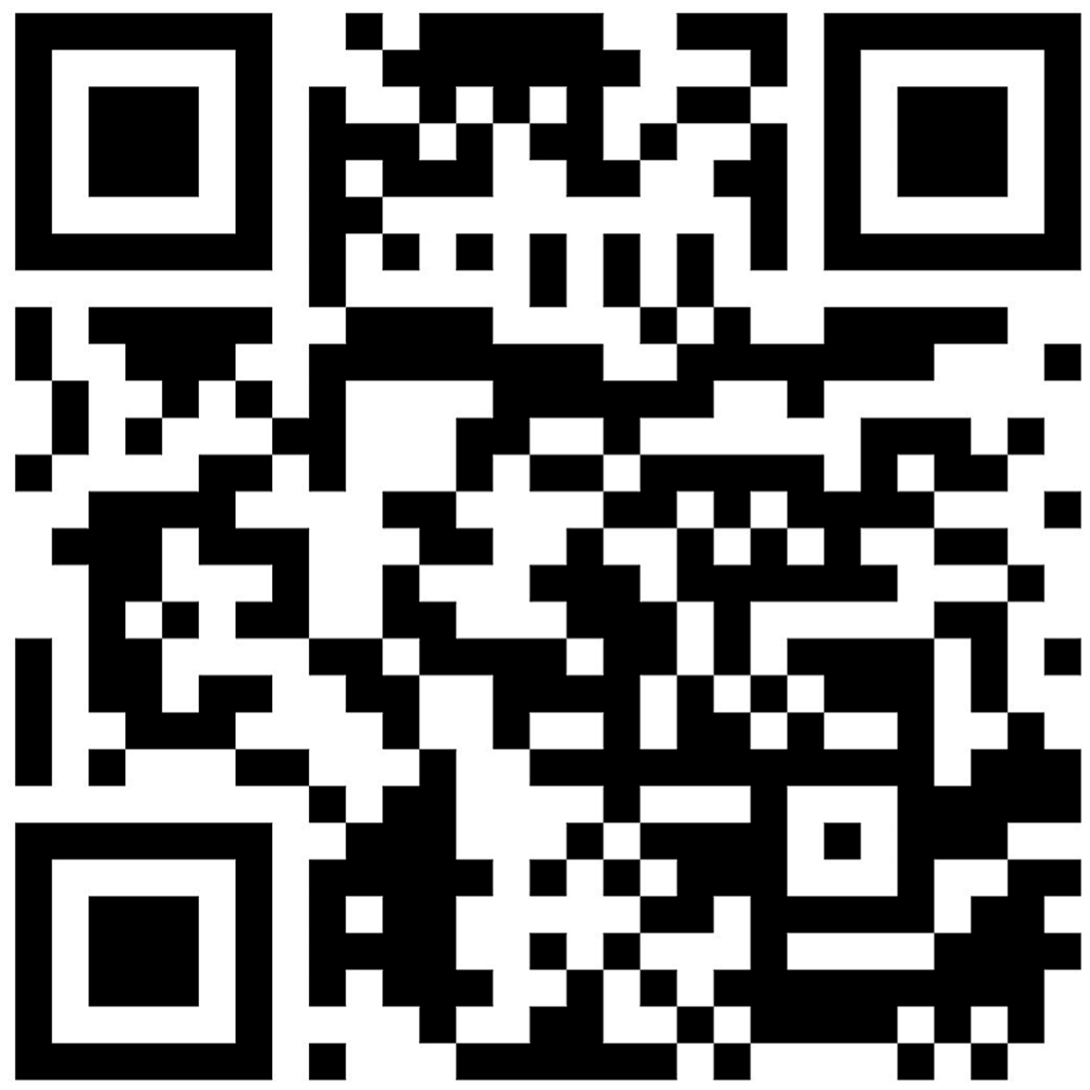
QR code linking to TEFE test.

A standardized version of the TEFE is available via the QR code provided below (Figure 2) to support consistent administration and facilitate future research and clinical application.

Access the TEFE (standardized version).

© 2026 April Wiechmann, PhD. All rights reserved.

This test may be used for clinical and educational purposes only. No modifications, redistribution, or commercial use permitted without written permission.

For authorized use and updates, access via official QR code or contact: April.Wiechmann@unthealth.edu

## Data Availability

The data analyzed in this study is subject to the following licenses/restrictions: The dataset analyzed in this study are not publicly available because they contain patient data and are subject to institutional privacy and ethical restrictions. De-identified data may be made available by the corresponding author upon request and with appropriate institutional approval. Requests to access these datasets should be directed to April.Wiechmann@unthealth.edu.

## References

[ref1] Amjad H. Roth D. L. Samus Q. M. Yasar S. Wolff J. L. (2016). Potentially unsafe activities and living conditions of older adults with dementia. J. Am. Geriatr. Soc. 64, 1223–1232. doi: 10.1111/jgs.14164, 27253366 PMC4914464

[ref2] Amjad H. Roth D. L. Sheehan O. C. Lyketsos C. G. Wolff J. L. Samus Q. M. (2018). Underdiagnosis of dementia: an observational study of patterns in diagnosis and awareness in US older adults. J. Gen. Intern. Med. 33, 1131–1138. doi: 10.1007/s11606-018-4377-y, 29508259 PMC6025653

[ref3] Bradford A. Kunik M. E. Schulz P. Williams S. P. Singh H. (2009). Missed and delayed diagnosis of dementia in primary care: prevalence and contributing factors. Alzheimer Dis. Assoc. Disord. 23, 306–314. doi: 10.1097/WAD.0b013e3181a6bebc, 19568149 PMC2787842

[ref4] Chatzidimitriou E. Ioannidis P. Moraitou D. Konstantinopoulou E. Aretouli E. (2023). The cognitive and behavioral correlates of functional status in patients with frontotemporal dementia: a pilot study. Front. Hum. Neurosci. 17:1087765. doi: 10.3389/fnhum.2023.1087765, 36923586 PMC10009888

[ref5] Folstein M. F. Folstein S. E. Fanjiang G. Psychological Assessment Resources Inc. (2001). Mini-Mental State Examination: Clinical Guide. Lutz, FL: Psychological Assessment Resources Inc.

[ref6] Forsund L. H. Grov E. K. Helvik A. S. Juvet L. K. Skovdahl K. Eriksen S. (2018). The experience of lived space in persons with dementia: a systematic meta-synthesis. BMC Geriatr. 18:33. doi: 10.1186/s12877-018-0728-0, 29390970 PMC5795848

[ref7] Gettel C. J. Chen K. Goldberg E. M. (2021). Dementia care, fall detection, and ambient-assisted living technologies help older adults age in place: a scoping review. J. Appl. Gerontol. 40, 1893–1902. doi: 10.1177/07334648211005868, 33853428 PMC8514579

[ref8] Iverson D. J. Gronseth G. S. Reger M. A. Classen S. Dubinsky R. M. Rizzo M. . (2010). Practice parameter update: evaluation and management of driving risk in dementia: report of the quality standards Subcommittee of the American Academy of neurology. Neurology 74, 1316–1324. doi: 10.1212/WNL.0b013e3181da3b0f, 20385882 PMC2860481

[ref9] Kotagal V. Langa K. M. Plassman B. L. Fisher G. G. Giordani B. J. Wallace R. B. . (2015). Factors associated with cognitive evaluations in the United States. Neurology 84, 64–71. doi: 10.1212/WNL.0000000000001096, 25428689 PMC4336093

[ref10] MacPherson S. E. Allerhand M. Cox S. R. Deary I. J. (2019). Individual differences in cognitive processes underlying trail making test-B performance in old age: the Lothian birth cohort 1936. Intelligence 75, 23–32. doi: 10.1016/j.intell.2019.04.001, 31293282 PMC6588265

[ref11] Marshall G. A. Rentz D. M. Frey M. T. Locascio J. J. Johnson K. A. Sperling R. A. . (2011). Executive function and instrumental activities of daily living in mild cognitive impairment and Alzheimer’s disease. Alzheimers Dement. 7, 300–308. doi: 10.1016/j.jalz.2010.04.005, 21575871 PMC3096844

[ref12] McDougall G. J. Becker H. Vaughan P. W. Acee T. W. Delville C. L. (2010). The revised direct assessment of functional status for independent older adults. Gerontologist 50, 363–370. doi: 10.1093/geront/gnp139, 19808842 PMC2867495

[ref13] Mioshi E. Hodges J. R. (2009). Rate of change of functional abilities in frontotemporal dementia. Dement. Geriatr. Cogn. Disord. 28, 419–426. doi: 10.1159/000255652, 19907178

[ref14] Mioshi E. Kipps C. M. Dawson K. Mitchell J. Graham A. Hodges J. R. (2007). Activities of daily living in frontotemporal dementia and Alzheimer disease. Neurology 68, 2077–2084. doi: 10.1212/01.wnl.0000264897.13722.53, 17562828

[ref15] Miyake A. Friedman N. P. (2012). The nature and organization of individual differences in executive functions: four general conclusions. Curr. Dir. Psychol. Sci. 21, 8–14. doi: 10.1177/0963721411429458, 22773897 PMC3388901

[ref16] Nyenhuis D. L. Reckow J. (2023). Office- and bedside-based screening for cognitive impairment and the dementias: which tools to use, interpreting the results, and what are the next steps? Clin. Geriatr. Med. 39, 15–25. doi: 10.1016/j.cger.2022.07.002, 36404027

[ref17] Royall D. R. Lauterbach E. C. Kaufer D. Malloy P. Coburn K. L. Black K. J. (2007). The cognitive correlates of functional status: a review from the Committee on Research of the American Neuropsychiatric Association. J. Neuropsychiatry Clin. Neurosci. 19, 249–265. doi: 10.1176/jnp.2007.19.3.249, 17827410

[ref18] Salis F. Costaggiu D. Mandas A. (2023). Mini-mental state examination: optimal cut-off levels for mild and severe cognitive impairment. Geriatrics 8:12. doi: 10.3390/geriatrics8010012, 36648917 PMC9844353

[ref19] Siddiqui M. Nyahoda T. Traber C. Elliott S. Wang V. Toledo-Franco L. . (2023). Screening for cognitive impairment in primary care: rationale and tools. Mo. Med. 120, 431–439. 38144923 PMC10743330

[ref20] Tierney M. C. Charles J. Naglie G. Jaglal S. Kiss A. Fisher R. H. (2004). Risk factors for harm in cognitively impaired seniors who live alone: a prospective study. J. Am. Geriatr. Soc. 52, 1435–1441. doi: 10.1111/j.0002-8614.2004.52404.x, 15341543

[ref21] Wiechmann A. Hall J. Azimipour S. (2015). Test of executive functioning in an emergency (TEFE): a performance-based assessment of safety for geriatric patients with dementia. Psychol. Neurosci. 8, 488–494. doi: 10.1037/pne0000030

